# Prevalence of pre-obesity and above and its associated factors in adult women: an analysis of the 2020 Korea National Health and Nutrition Examination Survey

**DOI:** 10.4069/whn.2024.05.21.1

**Published:** 2024-06-28

**Authors:** Hyunju Chae

**Affiliations:** College of Nursing, Sangmyung University, Cheonan, Korea

**Keywords:** Adult, Obesity, Overweight, Women

## Abstract

**Purpose:**

This study was conducted to determine the prevalence of pre-obesity (overweight) and above in adult women and to identify associated factors.

**Methods:**

Data were obtained from the eighth Korea National Health and Nutrition Examination Survey (KNHANES VIII-2), conducted in 2020. The sample comprised 2,288 women aged 19–64 years who participated in the KNHANES VIII-2. Data were analyzed using complex sample design analysis with SPSS version 20.1.

**Results:**

The prevalence of pre-obesity and above among adult women was 46.5%, with 18.6% classified as having pre-obesity and 27.9% as having obesity. A higher prevalence of pre-obesity and above was observed in women aged 50–59 years (odds ratio [OR]=1.67, *p*=.019) or 60–64 years (OR=1.80, *p*=.029); women whose highest educational attainment was high school (OR=1.28, *p*=.018) or middle school or less (OR=1.60, *p*=.017); those in middle-income households (OR=1.55, *p*=.005); those engaging in muscle-strengthening activities less than 2 days per week (OR=1.37, *p*=.019); and those sleeping less than 6 hours per night during the week (OR=1.37, *p*=.025).

**Conclusion:**

As nearly half of all adult women have either pre-obesity or obesity, prevention and management strategies must target both groups. Interventions should be prioritized for women in their 50s and older, as well as those with low education or income levels. Additionally, receiving adequate sleep of 7 hours or more and engaging in muscle-strengthening activities at least 2 days per week are important components of obesity management.

## Introduction

Pre-obesity is characterized by the accumulation of excess fat, while obesity is the accumulation of excess fat to the point of compromising health [1]. Both pre-obesity and obesity represent growing public health problems worldwide [1,2]. These conditions are major risk factors for various non-communicable diseases, including diabetes, cardiovascular disease, musculoskeletal disease, sleep apnea, depression, and cancer [3,4]. They are also associated with chronic health conditions and disabilities [5] and ultimately contribute to mortality [6]. Furthermore, pre-obesity and obesity cause substantial economic losses due to direct costs, such as medical expenses, and indirect costs, including those associated with premature death [7]. They also substantially impact labor productivity through high rates of absenteeism and low job satisfaction [2].

Pre-obesity and obesity rates are increasing worldwide [2], along with associated diseases [8]. The World Health Organization (WHO) reported that in 2016, 39% of adults globally had pre-obesity, while 13% had obesity [1]. Based on data from 16 member countries between 2017 and 2021, the Organization for Economic Co-operation and Development found that 34.3% and 25.7% of adults exhibited pre-obesity and obesity, respectively [3]. In Korea, data from the 2019 Korea National Health and Nutrition Examination Survey (KNHANES) indicated that 22.5% of adults had pre-obesity and 34.4% had obesity, corresponding to over half of the adult population exhibiting pre-obesity or above [9].

Reducing weight in adults with pre-obesity is a key step in preventing obesity, as the former often precedes the latter, and it becomes more challenging to normalize weight as pre-obesity progresses [10]. Although previous research has associated pre-obesity with a lower mortality risk compared to normal body mass index (BMI), recent meta-analyses and prospective observational studies have reported that mortality risk increases when BMI exceeds the normal range [11]. Furthermore, pre-obesity confers an elevated risk of hypertension and cardiovascular disease [12,13], and this risk is heightened when pre-obesity begins at an earlier age [12]. The Korean Society for the Study of Obesity (KSSO) recommends maintaining a BMI of less than 23 kg/m^2^. This recommendation is based on a national survey involving over 20 million participants in National Health Insurance Service health checkups, which revealed that individuals with a BMI of 23 kg/m^2^ or higher were more likely to have at least one of three adult diseases: diabetes, hypertension, or dyslipidemia [14]. There is insufficient awareness about the risks and severity of pre-obesity. While obesity is often addressed therapeutically, its associated health issues indicate the need for effective management of pre-obesity as a preventive measure, rather than solely focusing on the treatment of obesity [13]. In 2018, the KSSO updated its relevant guidance, categorizing obesity into stages 1, 2, and 3. Additionally, the guidelines replaced the term “overweight” with “pre-obesity” to underscore the associated risk of obesity [14].

Pre-obesity and obesity pose serious health risks and require regular monitoring [15]. To curb the growing obesity epidemic, it is necessary to focus on contributing situational or behavioral factors [2] and to identify variables linked to pre-obesity and obesity [16], as well as those associated with prevalence trends [15]. While many international studies have examined the prevalence and associated factors of both pre-obesity and obesity [2,15], most research conducted in Korea has focused exclusively on obesity [17]. Furthermore, when exploring factors related to obesity in domestic research, the focus is often on adolescents or the entire adult population [17]. However, the pathophysiology of obesity and the accumulation of visceral fat differs between women and men [8], and female-specific factors such as childbearing, pregnancy, and menopause impact obesity [8,18]. Therefore, it is imperative to identify factors relevant to obesity separately for women and men.

While the combined prevalence of pre-obesity and obesity is reportedly lower in women than in men [3], women face a higher risk of developing related diseases [7]. Additionally, obesity-associated healthcare costs appear to be higher for women than for men [19]. Women with obesity are also at increased risk of certain women’s health conditions, including irregular menstrual cycles, cancers such as endometrial and breast cancer, and pelvic disorders [18]. Moreover, in a society that values appearance and thinness, the negative perceptions fueled by prejudice and stereotypes contribute to depression among women with obesity [18,19]. Obesity-related stigma has also been described as having a more detrimental impact on women with the condition compared to their male counterparts [20]. Therefore, this study examined the prevalence and associated factors of pre-obesity abnormalities in adult women, including both individuals with obesity and those with pre-obesity. This research may provide a foundation for developing interventions for the prevention and management of obesity in adult women.

The purpose of this study is to determine the prevalence of pre-obesity and above in adult women, as well as to identify associated factors. The specific research objectives are as follows:

1) to ascertain the prevalence of pre-obesity and above among adult women;

2) to identify differences in this prevalence based on general characteristics;

3) to identify differences in this prevalence based on health-related characteristics; and

4) to identify factors associated with this prevalence among the study population.

## Methods

**Ethics statement:** The requirement for obtaining informed consent was waived by the Institutional Review Board of Joongbu University (JIRB-2023052201-01), as the study did not involve any sensitive information and the survey was conducted anonymously.

### Study design

This study presents a secondary analysis of data from the eighth KNHANES, conducted in 2020, to examine the prevalence of pre-obesity and above among adult women. Employing a descriptive correlational design, the analysis utilized the 2020 KNHANES dataset to ascertain this prevalence and to identify related factors in this population.

### Sample and sampling

**Figure 1.**
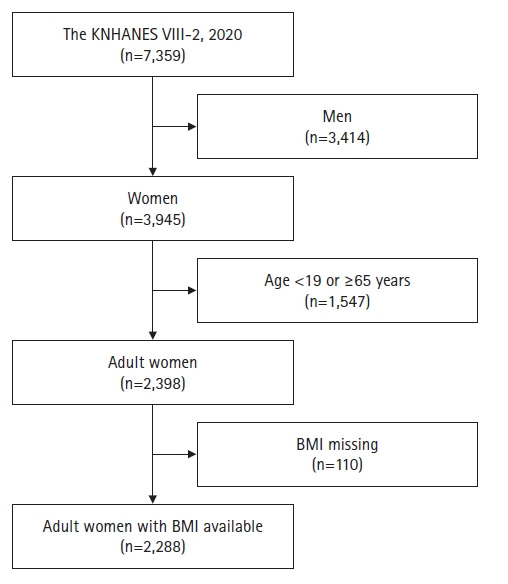
Flowchart of the study population. KNHANES VIII-2, the eighth Korea National Health and Nutritional Examination Survey; BMI, body mass index.

This study included adult women aged 19-64 years who participated in the KNHANES, conducted by the Korea Disease Control and Prevention Agency (KDCA), between January and December 2020. The categorization of pre-obesity and obesity within this study relied on BMI measurements; therefore, only adult women with available height and weight data were included. Of the 7,359 individuals who participated in the 2020 KNHANES, 3,945 were women. Among these, 2,398 were adult women within the specified age range, and 2,288 of them had complete height and weight data (Figure 1).

### Variables

#### Pre-obesity and above

In this study, BMI values were calculated using weight and height data from the KNHANES to define pre-obesity. According to the KSSO, BMI classifications are as follows: underweight, <18.5 kg/m^2^; normal, 18.5–22.9 kg/m^2^; pre-obesity, 23–24.9 kg/m^2^; obesity stage 1, 25–29.9 kg/m^2^; obesity stage 2, 30–34.9 kg/m^2^; and obesity stage 3, ≥35 kg/m^2^ [21]. For the purposes of this study, “pre-obesity and above” encompasses both pre-obesity and obesity, defined as a BMI of 23 kg/m^2^ or higher.

#### General characteristics

For general characteristics, data were acquired from the KNHANES on age, education reclassification, household income quintile, marital status, household type, head of household status, and occupation reclassification (employment status). Age was divided into five categories: 19–29, 30–39, 40–49, 50–59, and 60–64 years. Education was classified according to the highest level of attainment: less than high school graduation, high school graduation, or college graduation or higher. This system was based on reclassification of categories that included “less than middle school diploma,” “middle school diploma,” “high school diploma,” and “college diploma or higher.” Income levels were determined using household income quintiles—low, middle-low, middle, middle-high, and high—based on the average monthly household equivalized income. Marital status was categorized as unmarried or married. Household type was differentiated into one-person and multi-person households; the category “one family member in one household” was considered a one-person household, while all others were classified as multi-person households. Household head status was identified as a yes-or-no item using the relevant data. Finally, employment status was categorized based on occupational reclassification data, with individuals classified as either having a job (yes) or not having one (no).

#### Health-related characteristics

The health-related characteristics examined in this study included perceived stress, current smoking status, frequency of alcohol consumption and number of drinks consumed annually, aerobic physical activity, days of strength training per week, and average hours of sleep per night on weekdays and weekends. Perceived stress was classified into two categories. Stress perceived (yes) was associated with the responses “I feel very stressed” and “I tend to feel very stressed,” while stress unperceived (no) was indicated by either “I feel a little stressed” or “I rarely feel stressed.” Smoking status was categorized as smoking (yes) for individuals who reported smoking “daily” or “sometimes” and non-smoking (no) for those who had “smoked in the past but not currently” or had “never smoked.” Alcohol consumption was categorized as non-drinking for those who reported not drinking in the past year; high-risk drinking for individuals consuming an average of 5 or more drinks per occasion and drinking twice a week, as per the KNHANES criteria for high-risk drinking [22]; and non–high-risk drinking for the remaining respondents. Physical activity was assessed based on the prevalence of aerobic physical activity from the KNHANES, categorizing individuals as engaging in aerobic physical activity (yes) or not (no). This was determined by whether they participated in moderate-intensity physical activity for at least 2 hours and 30 minutes per week, vigorous-intensity physical activity for at least 1 hour and 15 minutes per week, or an equivalent combination of these. Muscle-strengthening activity was categorized based on WHO recommendations [23] as less than 2 days per week (no) or 2 or more days per week (yes), using data on the frequency of strength training. Finally, based on previous research, sleep duration was categorized as 6 hours or less, 7–8 hours, or 9 hours or more [24]. This categorization used open-ended average sleep duration per day, calculated for weekdays and weekends.

### Data collection and analysis

The data for this study were obtained from the KNHANES section of the KDCA website in 2020, following the completion of the Statistical Data User Compliance Pledge and the registration of user information. In accordance with the Personal Information Protection Act and the Statistics Act, the KDCA provides only de-identified data to prevent the identification of individuals from the survey data. The downloaded data were analyzed using IBM SPSS ver. 21.0 (IBM Corp., Armonk, NY, USA), considering the strata, clusters, and weights inherent in the design for a composite sample analysis. The specific analytical methods used were as follows.

1) The prevalence of pre-obesity and above among adult women was assessed using composite sample frequency analysis.

2) Differences in this prevalence within the study population, based on general and health-related characteristics, were examined using the Rao-Scott chi-square test.

3) Factors associated with the prevalence of pre-obesity and above were analyzed using multiple logistic regression.

## Results

### Prevalence of pre-obesity and above in adult women

Of the adult women, 18.6% had pre-obesity and 27.9% had obesity; thus, the combined prevalence of pre-obesity and above was 46.5% (Table 1).

### Variations in the prevalence of pre-obesity and above based on general and health-related characteristics in adult women

The prevalence of pre-obesity and above among adult women varied according to age, education, marital status, income, and head of household status. Increasing age was associated with a higher prevalence of pre-obesity and above (*χ*^2^=82.04, *p*<.001), as was lower education level (*χ*^2^=52.27, *p*<.001). Regarding income-based differences, this prevalence was higher in women with low, middle-low, and middle incomes compared to those with high incomes (*χ*^2^=24.00, *p*=.004). Married women had a higher prevalence of pre-obesity and above than unmarried women (*χ*^2^=47.56, *p*<.001), and household heads also had a higher prevalence than non-heads of household (*χ*^2^=10.71, *p*=.006) (Table 2).

The prevalence of pre-obesity and obesity in the study population also varied according to health-related characteristics, including the frequency of muscle-strengthening activity and weekday and weekend sleep durations. Women who engaged in muscle-strengthening activities less than 2 days per week exhibited a higher prevalence of pre-obesity and obesity compared to those who did not (*χ*^2^=11.80, *p*=.002). Furthermore, the highest prevalence rates were observed in women who slept 6 hours or less on weekdays (*χ*^2^=11.92, *p*=.012) and weekends (*χ*^2^=12.21, *p*=.007), relative to those who slept 7 to 8 hours (Table 2).

### Factors associated with the prevalence of pre-obesity and above in adult women

The prevalence of pre-obesity and above among adult women was associated with age, education level, income, muscle-strengthening activity, and weekday sleep duration. Specifically, the prevalence was 1.67 times higher (*p*=.019) in women aged 50–59 years and 1.80 times higher (*p*=.029) in women aged 60–64 years, compared to those 19–29 years old. Women with only a high school diploma were 1.28 times more likely (*p*=.018) to have pre-obesity or obesity than those with a college degree or higher, and this likelihood rose to 1.60 (*p*=.017) for women with less than a high school education. Additionally, women with middle incomes displayed a 1.55 times greater prevalence (*p*=.005) of pre-obesity or obesity compared to those with high incomes. Engaging in muscle-strengthening activities less than 2 days per week was associated with a 1.37-fold higher prevalence (*p*=.019) than more frequent activity, and women who slept 6 hours or less per day similarly had a 1.37-fold higher prevalence (*p*=.025) compared to those sleeping 7 to 8 hours per day (Table 3).

## Discussion

This study conducted a secondary analysis of data from the 2020 KNHANES to determine the prevalence of pre-obesity and obesity in adult women, as well as to identify factors associated with these conditions.

Overall, 18.6% of the participants had pre-obesity and 27.9% had obesity; thus, the combined prevalence was 46.5%. This figure is lower than the 56.2% prevalence of pre-obesity and above reported among Iranian women [25], yet higher than the 43.2% reported among Botswana women [26]. Analysis of data from the 2019 KNHANES indicated that the prevalence of pre-obesity and obesity among adult women aged 19 years and older was 46.4%, with 19.1% exhibiting pre-obesity and 27.3% obesity. Among young adult women (19–39 years), the rate was 30.0% (pre-obesity, 11.0%; obesity, 19.0%), and among middle-aged women (40–64 years), the rate was 50.8% (pre-obesity, 21.7%; obesity, 29.1%) [9]. Considering the 2019 KNHANES data [9] and the findings of this study, nearly half of Korean adult women aged 19-64 have at least pre-obesity, with about half of this group in the obese category. These findings underscore the urgency of obesity prevention and management, targeting women with pre-obesity as a group at high risk. Consequently, there is a need to broaden the scope of obesity prevention and management to include this group. Additionally, more practical and effective prevention and management programs should be developed, informed by ongoing research into factors contributing to the prevalence of pre-obesity and obesity in adult women.

In this study, the prevalence of pre-obesity and above was higher in adult women aged 50–59 and 60–64 years compared to those aged 19–29 years. This aligns with previous research [16,25] indicating an increased prevalence of pre-obesity and above as women age. However, some studies have found no association between age and obesity in women [17]. This discrepancy may stem from the fact that these studies only included women aged 19–39 years and compared the mean ages of women with and without obesity, rather than examining differences in obesity prevalence across age groups. Therefore, based on previous research [16,25] and our findings, the prevalence of pre-obesity and above tends to rise with age. The growing prevalence of pre-obesity and above among aging women may be attributed to a decline in physical activity and an increased consumption of calorie-dense foods, such as sugary snacks [27]. A study on Korean adults found that the consumption of high-sodium foods, such as soups, stews, and stewed dishes, escalated with age among those with pre-obesity and above [28]. Given that high sodium intake has been linked to an elevated risk of obesity [29], the rising prevalence of pre-obesity and above in Korean adult women could be associated with such unhealthy dietary habits. Therefore, to minimize the prevalence of pre-obesity and obesity, it is essential to promote lifestyle changes that encourage physical activity and to provide education on healthy eating practices, including the reduction of salty food consumption. Additionally, active interventions by nutrition professionals are necessary [16,28].

In this study, the prevalence of pre-obesity and above was higher among adult women with lower educational levels. This finding aligns with previous research indicating that lower education is associated with a higher prevalence of pre-obesity and above [25] as well as obesity alone [30,31], and it challenges studies suggesting that a higher educational level correlates with increased rates of pre-obesity and above [16]. Additionally, the prevalence of pre-obesity and above was greater among those with middle income compared to those with high income. This observation is consistent with studies showing that lower income is linked to a higher prevalence of pre-obesity and above [32] and obesity [30,31], while contradicting separate findings regarding pre-obesity and above [16]. Given that obesity rates differ by socioeconomic status across countries, research on Korean women indicates that the higher prevalence of obesity among those with lower education and income levels reflects the socioeconomic development in Korea [30]. Therefore, based on the results of this study and previous research, Korean women with lower education and income levels are more likely to experience pre-obesity or obesity. Women with higher educational levels tend to adopt healthier lifestyles and eating habits, as they possess greater knowledge about health and nutrition and are more cognizant of the consequences of obesity [26]. Furthermore, income influences health behaviors such as healthy eating and participation in weight control programs, with higher income levels offering fewer restrictions on health behavior choices [33]. Consequently, it is imperative to raise awareness about the risks associated with pre-obesity and obesity and to implement education and mass media campaigns that promote healthy lifestyles and eating habits. Additionally, providing free programs for low-income individuals and increasing economic support at the community or national level are necessary steps to address these health concerns.

In the present study, the prevalence of pre-obesity and above was lower in adult women who engaged in muscle-strengthening activities at least 2 days per week compared to those who did not. This finding aligns with research [34] indicating that engaging in muscle-strengthening activities at this frequency is linked to a lower body fat percentage. However, our study contrasts with earlier research [17] suggesting no association between muscle-strengthening activities and obesity. This discrepancy may stem from differences in the study populations; our research encompassed women aged 19–64 years, while the prior study [17] was limited to those aged 19–39 years. Exercise, including aerobic and strength training, is associated with changes in body composition and weight loss [35,36]. Strength training, in particular, has been shown to be more effective for fat loss in individuals with pre-obesity [35,37]. Consequently, it is advisable for women to participate in muscle-strengthening activities at least 2 days per week to prevent and manage obesity. Despite these benefits, it has been reported that women are less likely than men to engage in muscle-strengthening activities [38]. Therefore, there is a need for strategies to increase participation among women, such as providing education about the benefits of strength training, raising awareness, and administering simple and accessible strength training programs. Moreover, there is a relative scarcity of research on the relationship between muscle-strengthening activities and obesity compared to aerobic exercise [39]. Therefore, further studies are necessary to explore the relationship between the type, intensity, and frequency of muscle-strengthening activities and obesity. Based on the findings of such research, a variety of strength training programs should be developed and offered.

In this study, the prevalence of pre-obesity and above was higher in adult women who slept 6 or fewer hours per night compared to those who slept 7 to 8 hours. This observation aligns with previous research [40,41] indicating a greater prevalence of pre-obesity and obesity among women with shorter sleep durations. A meta-analysis examining the link between sleep duration and health outcomes revealed that short sleep is associated with obesity [42]. Furthermore, epidemiological evidence suggests a relationship between reduced sleep duration and increased rates of pre-obesity and obesity, both cross-sectionally and longitudinally [43]. Sleep deprivation can lead to increased hunger by elevating levels of the appetite-stimulating hormone ghrelin, which, in turn, can lead to increased food intake and contribute to obesity [44]. From a behavioral perspective, engaging in less sleep may also heighten obesity risk by extending the period of wakefulness, which can result in increased food consumption [41]. Consequently, ensuring adequate sleep is essential for obesity prevention, and a range of strategies, including education and promotion, should be implemented.

This study examined the prevalence of pre-obesity and above, as well as associated factors, in adult women by analyzing data from the KNHANES, a nationally representative dataset. However, our analysis did not account for dietary variables when examining associated factors. Consequently, future research should incorporate these variables to better identify related factors. Moreover, as our study was limited to adult women aged 19–64 years, subsequent studies should include older women. This would enable a more extensive understanding of the prevalence of pre-obesity and obesity, as well as associated factors, across age groups, which is crucial for effective obesity prevention and management throughout a woman’s life. Lastly, while this study collectively addressed pre-obesity and obesity, future research should differentiate between the two groups. Separate analysis will help to elucidate the differences in prevalence and associated factors between these populations.

Despite these limitations, the study is meaningful in that it extends beyond obesity to determine the prevalence of pre-obesity and above, as well as its associated factors, in adult women. Nearly half of the adult women in this study exhibited pre-obesity or above, underscoring the importance of including pre-obesity in obesity prevention and management strategies. Additionally, the findings provide a basis for comparison of changes in the prevalence or associated factors of pre-obesity and above in future research.

In conclusion, since nearly half of adult women fall into the pre-obesity category or higher, it is essential to include women with pre-obesity in obesity prevention and management strategies. Priority should be given to women aged 50 years and above, as well as those with lower levels of education and income. Additionally, maintaining a sleep duration of at least 7 hours and engaging in muscle-strengthening activities a minimum of 2 days per week are necessary measures.

## Figures and Tables

**Table 1.** Prevalence of pre-obesity and above in adult women (N=2,288)

Categories	Body mass index (kg/m^2^)	%^†^ (SE)
Underweight	<18.5	6.7 (0.7)
Normal	18.5–22.9	46.7 (1.4)
Pre-obesity	23.0–24.9	18.6 (1.0)
Obesity stage 1	25.0–29.9	21.9 (1.1)
Obesity stage 2	30–34.9	5.0 (0.6)
Obesity stage 3	≥35.0	1.0 (0.2)

† Weighted percentile.

**Table 2.** Prevalence of pre-obesity and above among women based on general and health-related characteristics (N=2,288)

Characteristic	Categories	n^†^	%^‡^ (SE)	%^‡^ (SE)		*χ*^2^ (*p*)
				BMI <23.0 kg/m^2^	BMI ≥23.0 kg/m^2^	
Age (years)	19–29	397	20.9 (1.1)	65.1 (3.0)	34.9 (3.0)	82.04 (<.001)
	30–39	417	19.3 (1.2)	61.8 (2.7)	38.2 (2.7)	
	40–49	545	23.5 (1.2)	53.0 (2.4)	47.0 (2.4)	
	50–59	579	25.0 (1.0)	45.0 (2.5)	55.0 (2.4)	
	60–64	350	11.2 (0.7)	37.2 (3.2)	62.8 (3.2)	
Education	Less than high school	316	11.1 (0.9)	35.3 (3.2)	64.7 (3.1)	52.27 (<.001)
	High school	861	41.1 (1.5)	50.7 (1.9)	49.3 (2.0)	
	College or higher	1,009	47.8 (1.7)	59.9 (1.8)	40.1 (1.8)	
Income	Low	150	6.1 (0.7)	44.5 (5.8)	55.5 (5.8)	24.00 (.004)
	Middle-low	317	13.2 (1.0)	48.4 (3.3)	51.6 (3.3)	
	Middle	533	23.2 (1.1)	48.8 (2.7)	51.2 (2.7)	
	Middle-high	636	28.2 (1.2)	55.1 (2.4)	44.9 (2.4)	
	High	648	29.3 (1.8)	59.9 (2.3)	40.1 (2.3)	
Marital status	Unmarried	529	26.7 (1.2)	65.4 (2.6)	34.6 (2.6)	47.56 (<.001)
	Married	1,759	73.3 (1.2)	49.1 (1.6)	50.9 (1.6)	
Household type	One-person	169	6.2 (0.8)	54.2 (4.9)	45.8 (4.9)	0.04 (.874)
	Multi-person	2,119	93.8 (0.8)	53.4 (1.5)	46.6 (1.5)	
Head of household	No	1,511	68.4 (1.2)	55.8 (1.8)	44.2 (1.8)	10.71 (.006)
	Yes	777	31.6 (1.2)	48.4 (2.1)	51.6 (2.1)	
Employed	No	891	40.0 (1.4)	52.8 (2.4)	47.2 (2.4)	0.25 (.695)
	Yes	1,397	60.0 (1.4)	53.9 (1.6)	46.1 (1.6)	
Perceived stress	No	1,530	67.0 (1.2)	54.2 (1.6)	45.8 (1.6)	0.67 (.523)
	Yes	749	33.0 (1.2)	52.3 (2.5)	47.7 (2.5)	
Smoking status	No	2,143	93.8 (0.6)	53.7 (1.5)	46.3 (1.5)	0.31 (.668)
	Yes	136	6.2 (0.6)	51.3 (5.3)	48.7 (5.3)	
Drinking status	Non-drinking	645	26.1 (1.1)	51.3 (2.4)	48.7 (2.4)	2.27 (.451)
	Non–high-risk drinking	1,488	67.5 (1.1)	54.6 (1.8)	45.4 (1.8)	
	High-risk drinking	146	6.4 (0.6)	51.3 (5.0)	48.7 (5.0)	
Physical activity	No	1,245	55.9 (1.3)	53.3 (1.8)	46.7 (1.8)	0.01 (.943)
	Yes	941	44.1 (1.3)	53.5 (2.0)	46.5 (2.0)	
Muscle-strengthening activity (days/week)	<2	1,822	82.9 (0.9)	51.7 (1.5)	48.3 (1.5)	11.80 (.002)
	≥2	364	17.1 (0.9)	61.5 (2.9)	38.5 (2.9)	
Weekday sleep duration (hours/day)	≤6	941	40.8 (1.0)	49.1 (2.0)	50.9 (2.0)	11.92 (.012)
	7-8	1,096	47.2 (1.1)	56.6 (1.9)	43.4 (1.9)	
	≥9	251	12.0 (0.8)	56.0 (3.7)	44.0 (3.7)	
Weekend sleep duration (hours/day)	≤6	595	24.6 (0.9)	47.4 (2.4)	52.6 (2.4)	12.21 (.007)
	7-8	1,089	47.0 (1.2)	54.5 (1.9)	45.5 (1.9)	
	≥9	604	28.4 (0.9)	57.0 (2.4)	43.0 (2.4)	

BMI, body mass index.

† Unweighted frequency,

‡ weighted percentile.

**Table 3.** Factors associated with pre-obesity and above in adult women (N=2,288)

Variable	Categories	Odds ratio (95% CI)	*p*
Age (year)^†^	60–64	1.80 (1.06–3.05)	.029
	50–59	1.67 (1.09–2.57)	.019
	40–49	1.27 (0.86–1.88)	.236
	30–39	0.94 (0.63–1.40)	.771
Education^†^	Less than high school	1.60 (1.09–2.35)	.017
	High school	1.28 (1.04–1.57)	.018
Income^†^	Low	1.63 (0.95–2.79)	.075
	Middle-low	1.40 (1.00–1.95)	.052
	Middle	1.55 (1.14–2.09)	.005
	Middle-high	1.14 (0.86–1.52)	.352
Marital status^†^	Married	1.39 (0.99–1.94)	.058
Head of household^†^	Yes	1.01 (0.80–1.27)	.942
Muscle-strengthening activity^†^ (days/week)	<2	1.37 (1.05–1.77)	.019
Weekday sleep duration^†^ (hours/day)	≤6	1.37 (1.04–1.79)	.025
	≥9	0.95 (0.60–1.52)	.830
Weekend sleep duration^†^ (hours/day)	≤6	0.92 (0.67–1.25)	.589
	≥9	1.11 (0.84–1.47)	.478

CI, confidence interval.

† The dummy variable references were age (19–29 years), education (college or higher), income (high), marital status (unmarried), head of household (no), muscle-strengthening activity (≥2 days/week), weekday sleep duration (7-8 hours/day), and weekend sleep duration (7-8 hours/day).
